# A brush with danger: operative management of a through and through urethral and rectal injury following paintbrush insertion into the urethra

**DOI:** 10.1308/rcsann.2025.0033

**Published:** 2025-08-26

**Authors:** R Karanjia, D Whiting, M Qureshi, H Dowson, A Chetwood

**Affiliations:** Frimley Health NHS Foundation Trust, UK

**Keywords:** Paintbrush, Urethral injury, Foreign body, Rectal injury, Sexual trauma

## Abstract

Iatrogenic injury from urethral foreign body insertion is rare but a recognised urological emergency. We present a case of a 61-year-old man who presented to the emergency department having inserted a 15cm-long paintbrush into his urethra, resulting in two ‘exit’ wounds in his urethra with an associated rectal injury. The patient was taken to theatre and a primary perineal repair of the two ‘exit’ defects in his urethra was performed over a urethral catheter and covering suprapubic catheter. His rectal injury was also repaired with a covering laparoscopic defunctioning colostomy performed. The patient recovered fully, without any residual urinary symptoms. To our knowledge, there are no reported cases of concomitant urethral and rectal injury following urethral foreign body insertion.

## Background

Young men are the patient demographic most affected by sexual-related trauma, with penile fracture during intercourse the most common injury.^1^ Less common injuries include penile strangulation from constriction devices, as well as urethrovesical foreign bodies (FBs), which are also more common in men.^2^ The operative management of penile fractures and penile constriction devices is well known and considered an essential skill for any urologist. However, the management of urethrovesical foreign bodies is more challenging, due to the myriad of FBs that can be inserted, as well as their relative rarity. Operative management is usually via endoscopic removal and catheterisation, although this can risk severe urosepsis and potential for fistula development.^3^ Definitive surgical repair via a perineal approach is also described.^4^

We report a case of a ‘through and through’ urethral injury, with concomitant rectal injury, in a 61-year-old man who inserted a 15cm-long paintbrush into his urethra during autoeroticism, which required definitive operative repair of both the urethra and rectum. At the time of writing, there are no reported cases on reconstructive repair of concomitant rectal and urethral injury following urethral FB insertion.

## Case history

**Figure 1:**
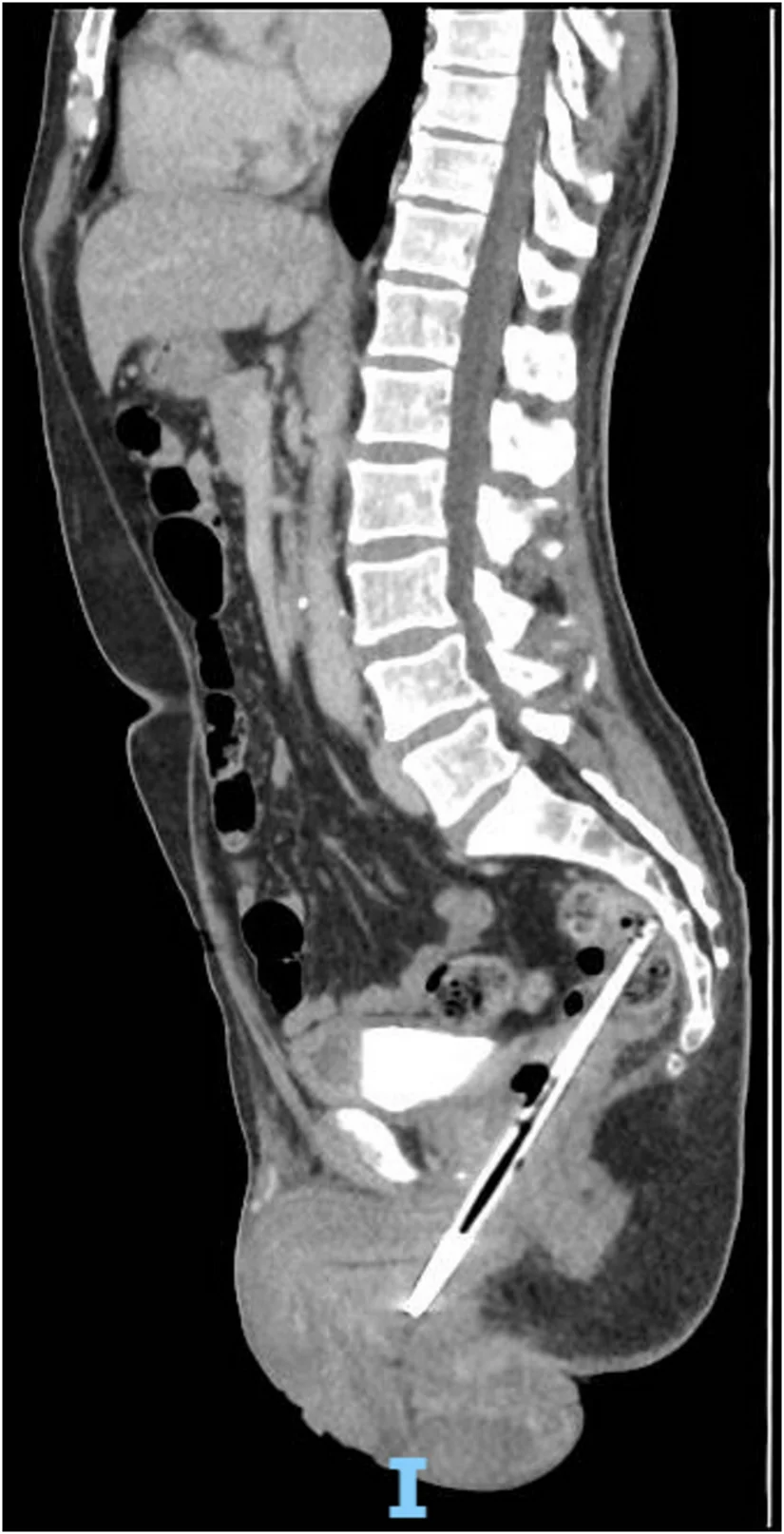
Sagittal CT slice demonstrating the paintbrush penetrating the bulbar urethral through the rectum and terminating at the rectosigmoid junction without perforation into the presacral space. CT = computed tomography

A 61-year-old man presented to the emergency department after inserting a paintbrush into his urethra, handle end first, during autoeroticism two days before, which he was unable to retrieve. He was not in urinary retention but had noticed urine leaking from his rectum. He had no significant past medical history and took no regular medications. The paintbrush could be palpated in the perineum and the handle was palpable inside the rectum on digital rectal examination. His observations and blood tests were stable, and the patient was not septic. He was commenced on broad spectrum IV antibiotics. A computed tomography (CT) scan confirmed the penetrating injury through the bulbar urethra into the rectum and was suspected to be entirely extraperitoneal (Figure 1). After discussion with the patient and the general surgical team, the patient was consented for a defunctioning colostomy, cystoscopy, urethral and rectal repair via a perineal approach and urinary diversion with suprapubic catheter (SPC).

**Figure 2:**
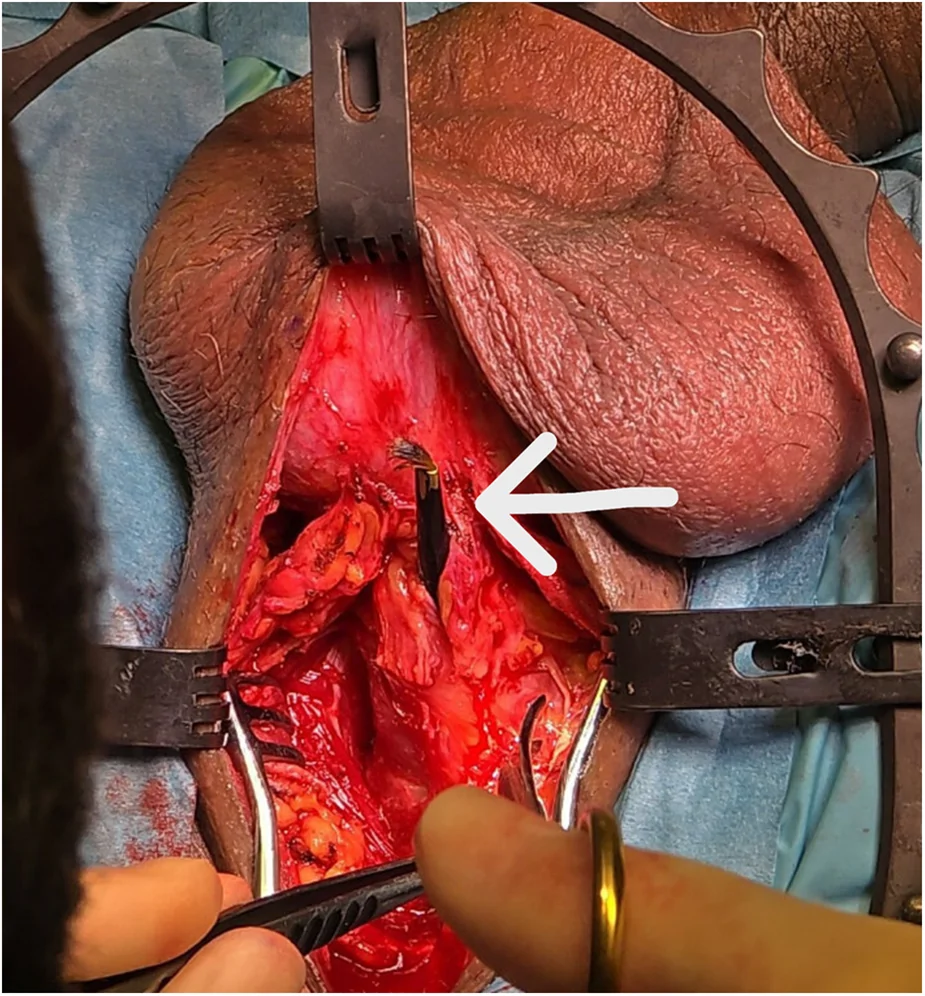
Brush end of the paintbrush identified at the penoscrotal junction in situ exiting the ventral urethra. This injury was thought to be caused by the pressure on the ventral urethra from its fixed position at the proximal (handle) end.

**Figure 3:**
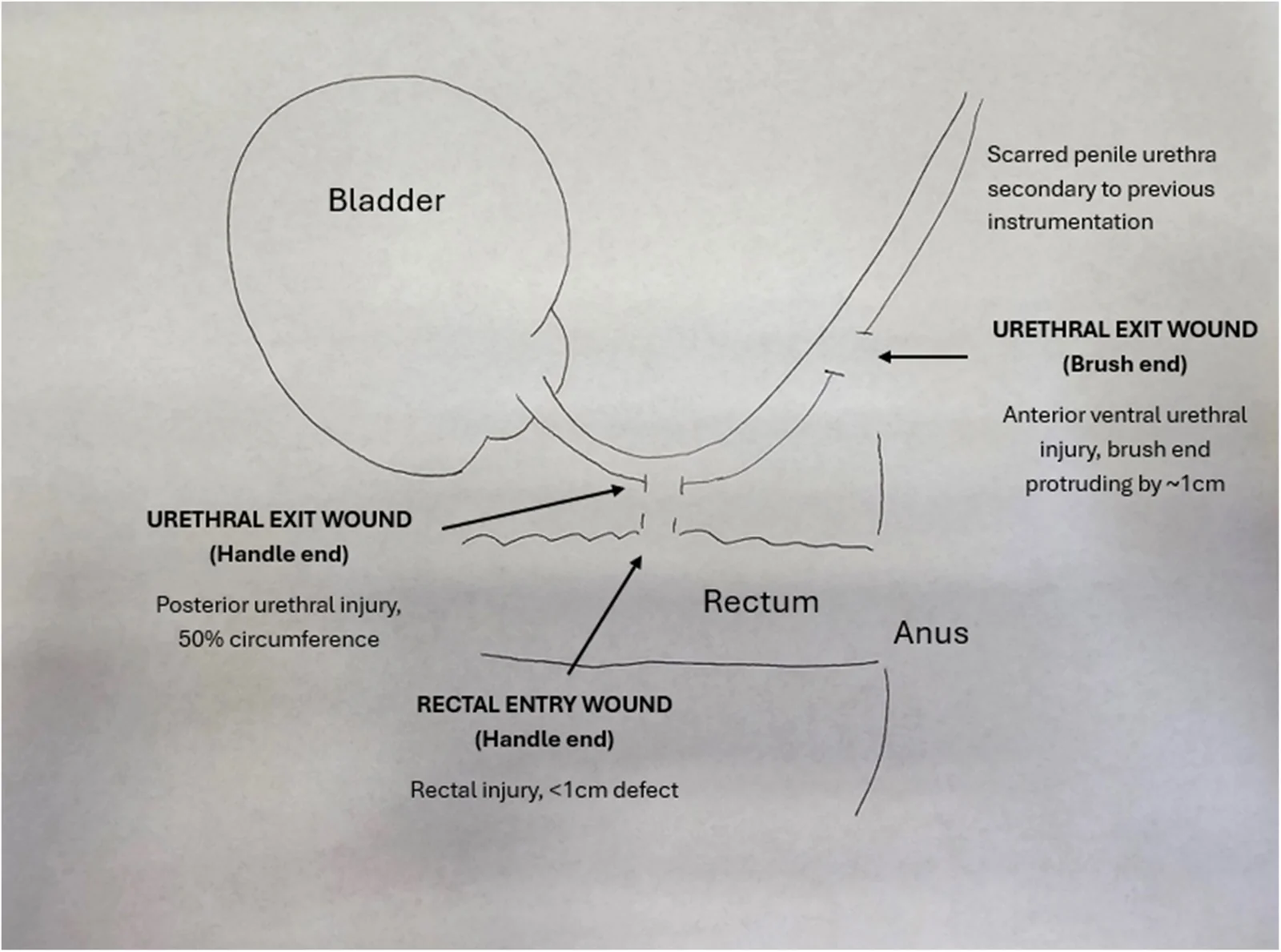
Intraoperative diagram demonstrating the two urethral ‘exit’ wounds of the paintbrush with the associated rectal ‘entry’ wound from the handle end.

General anaesthesia was given and the patient placed in lithotomy position. The patient was prepped and draped as per a bulbar urethroplasty. Cystoscopy confirmed penile scarring from previous urethral sounding. Attempts to manipulate the brush with biopsy forceps were unsuccessful. A midline Raphe incision was used to mobilise the urethra and identify the bulbospongiosus. Tissue was noted to be oedematous secondary to urine extravasation. The brush end of the paintbrush was identified, in situ, exiting the ventral urethra at the penoscrotal junction (Figure 2). It was thought likely the brush end had injured and then ‘exited’ the ventral urethra due to the pressure from its fixed position at the proximal (handle) end. A final operative diagram is included, demonstrating both urethral ‘exit’ injuries from each end of the paintbrush and the associated rectal injury (Figure 3).

**Figure 4:**
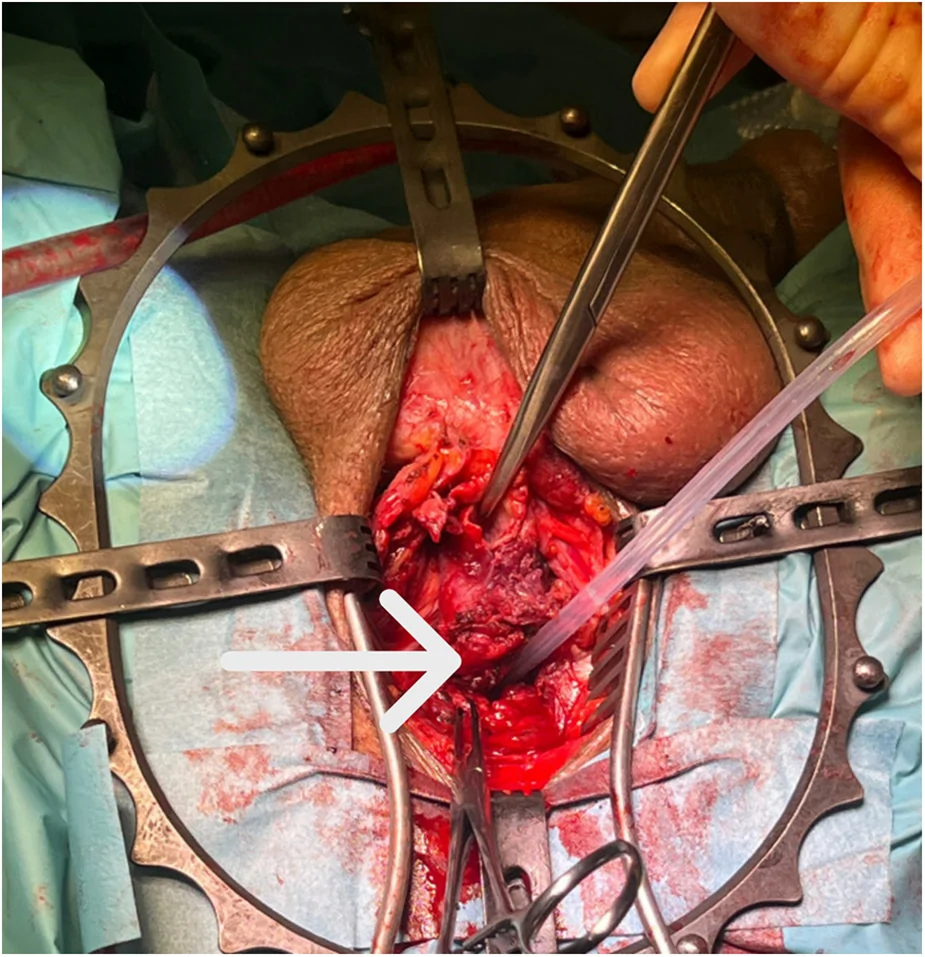
A catheter was placed into the proximal urethra to help confirm position and aid dissection.

The urethra was further mobilised to identify the site of the rectal perforation at the handle end. The FB provided a helpful marker to identify the urethral-rectal injury. Confirmation of the site of injury was helped by placing a finger in the rectum and using a catheter inserted into the proximal urethra (Figure 4).

Dissection continued until the edges of both the proximal bulbar urethral and rectal injuries were clearly identified. The rectum was closed with interrupted 2-0 vicryl in transverse fashion and the posterior urethral defect closed with interrupted 4-0 vicryl in vertical fashion. The ventral urethral injury was repaired using 4-0 vicryl after debridement of the edges of the injury. A perineal fat flap was manufactured from the left side and interposed between the two closure sites of the posterior urethral and rectal injuries to help prevent a recurrent fistula. The perineum was closed in three layers to cover the urethra and interrupted 2-0 prolene was used for the skin. A 10Fr Redivac drain was left and SPC inserted, in addition to a urethral catheter. A laparoscopic defunctioning colostomy was then performed by the colorectal team. Check flexible sigmoidoscopy confirmed no visible rectal injury.

The patient was kept on IV antibiotics for a further two days and the drain removed successfully at 48h. He was discharged home four days postoperatively. His sutures were removed and a pericatheter urethrogram three weeks postoperatively demonstrated no urine leak. The urethral catheter was removed and the SPC removed three weeks later. At the time of writing, the patient remains well, without urinary symptoms, and is awaiting colostomy reversal with the general surgeons.

## Discussion

This case highlights the risks of urethral foreign body insertion and the serious concomitant injuries that can occur. Patients often have delayed presentation due to embarrassment, which can impact on surgical repair and risks of sepsis.^4,5^ Subsequently, diagnosis may also not be straightforward. Flexible urethroscopy is the gold standard for direct visualisation of the urethra but may be poorly tolerated and may not fully appreciate adjacent injuries. CT is helpful for this reason, and can help detect any other FBs present, although image artefact may limit findings.

Choice of management will depend on many factors, including the nature of the FB, presence of concomitant injuries, systemic infection, patient comorbidities, presence of urinary retention and location in the genitourinary tract. In our case, the patient was fit and healthy, so definitive reconstruction was possible with the aim of minimising the risk of infection, urethral stricture or fistula in the future. However, in emergency situations, urinary and bowel diversion with SPC and defunctioning stoma may be the only option. The presence of a reconstructive urologist with the skill set to perform a primary repair is crucial and may not be available in all centres.

Many techniques have been described for urethral FB removal: open removal via perineal approach,^4^ endoscopic removal with cystoscopy and biopsy forceps,^6^ placing a catheter proximal to the FB and inflating it to milk the FB to the meatus^7^ or even conservative management for smaller FBs.^6^ In this case, the FB was unable to be retrieved endoscopically, and was lodged into the rectum, necessitating an open removal and repair. The technique described was performed by a surgeon with specialist interest in reconstructive urological surgery. Upon reflection, our learning points would be that leaving the paintbrush in situ helped the surgeon identify the entry and exit points on injury for the urethra, as well as the rectum, which helped facilitate definitive repair. Helpfully, the brush and handle end also allowed for orientation of the FB and injury identification.

## Conclusions

There are multiple methods described to remove urethral FBs, and a consideration of concomitant injuries, operative experience and patient comorbidities should be made before an operative decision is made. If concomitant injuries are suspected, cross-sectional imaging is essential, and repair should be performed by an experienced surgeon in conjunction with a general surgeon when appropriate.

## Consent for publication

A consent form was signed by the patient agreeing to use of their history and images for publication.
